# Lipid-Associated Variants near ANGPTL3 and LPL Show Parent-of-Origin Specific Effects on Blood Lipid Levels and Obesity

**DOI:** 10.3390/genes13010091

**Published:** 2021-12-29

**Authors:** Anna Lessmark, Gad Hatem, Györgyi Kovacs, Marta Vitai, Emma Ahlqvist, Tiinamaija Tuomi, Laszlo Koranyi, Leif Groop, Rashmi B. Prasad

**Affiliations:** 1 Lund University Diabetes Centre, Lund University, CRC, Skåne University Hospital Malmö, 214 28 Malmö, Sweden; 2 Genomics, Diabetes and Endocrinology, Department of Clinical Sciences, Lund University, CRC, 214 28 Malmö, Sweden; gad.hatem@med.lu.se (G.H.); emma.ahlqvist@med.lu.se (E.A.); leif.groop@med.lu.se (L.G.); 3 Heart Center Foundation, DRC, 8230 Balatonfured, Hungary; gyorgyi.kovacs@outlook.com (G.K.); marta.vitai@drc.hu (M.V.); laszlo.koranyi@drc.hu (L.K.); 4 Abdominal Center, Endocrinology, Helsinki University Central Hospital, Research Program for Clinical and Molecular Metabolism, University of Helsinki, 00260 Helsinki, Finland; tiinamaija.tuomi@hus.fi; 5 Institute for Molecular Medicine Finland (FIMM), University of Helsinki, 00290 Helsinki, Finland; 6 Folkhälsan Research Centre, 00250 Helsinki, Finland

**Keywords:** parent-of-origin, human genetics, dyslipidemia, obesity, ANGPTL3, LPL

## Abstract

Parent-of-origin effects (POE) and sex-specific parental effects have been reported for plasma lipid levels, and a strong relationship exists between dyslipidemia and obesity. We aim to explore whether genetic variants previously reported to have an association to lipid traits also show POE on blood lipid levels and obesity. Families from the Botnia cohort and the Hungarian Transdanubian Biobank (HTB) were genotyped for 12 SNPs, parental origin of alleles were inferred, and generalized estimating equations were modeled to assess parental-specific associations with lipid traits and obesity. POE were observed for the variants at the *TMEM57*, *DOCK7/ANGPTL3*, *LPL*, and *APOA* on lipid traits, the latter replicated in HTB. Sex-specific parental effects were also observed; variants at *ANGPTL3/DOCK7* showed POE on lipid traits and obesity in daughters only, while those at *LPL* and *TMEM57* showed POE on lipid traits in sons. Variants at *LPL* and *DOCK7/ANGPTL3* showed POE on obesity-related traits in Botnia and HTB, and POE effects on obesity were seen to a higher degree in daughters. This highlights the need to include analysis of POEs in genetic studies of complex traits.

## 1. Introduction

Dyslipidemia is one of the modifiable risk factors for cardiovascular disease (CVD) [1,2], which is the leading cause of death globally [3]. Dyslipidemias are common traits, with prevalence in the adult U.S. population being 43.4% for high total cholesterol (TC) (>200 mg/dL), 13.8% for high low-density lipoprotein-cholesterol (LDL-C) (>130 mg/dL), and 21.8% for low high-density lipoprotein-cholesterol (HDL-C) (<40 mg/dL) [4].

Plasma cholesterol levels are mediated by a complex interplay of genetic and environmental factors. There is also a strong genetic component, and >350 SNPs corresponding to >200 loci associated with circulating plasma lipids have been reported so far [5,6]. Currently, the heritability of plasma cholesterol levels has been estimated in several studies, with heritability of TC, HDL-C, and LDL-C level ranging from 0 to 89% [7,8,9,10,11], 22 to 93% [11,12,13,14], and 22 to 91% [7,11,14,15], respectively. In the Botnia study, in western Finland and southern Sweden, heritability estimates for TC ranged between 0.47–0.49, 0.52–0.61 for HDL-C, and 0.48–0.50 for LDL-C [16]. 

Sex of the parent also influences the offspring’s lipid levels, and these effects could also be different depending on the sex of the offspring. For instance, female offspring of diabetic mothers showing lower HDL-C than male offspring [17] and sex-specific parental effects were strongest for total cholesterol concentrations [16] in the Botnia families. Other studies have shown that male offspring of diabetic mothers had higher TG than daughters [18]. Additionally, female offspring of diabetic mothers were shown to have higher TC than female offspring of diabetic fathers [19]. A South-East Asian study showed an excess maternal transmission of obesity, insulin resistance, and dyslipidemia to offspring with one diabetic parent [20].

Some of these findings could be due to genetic parent-of-origin effects (POE), where an allele might have a different effect on a certain phenotype when inherited from one parent but be neutral or having opposite effect when inherited from the other. Some of this could be attributed to epigenetic mechanisms, such as preferential DNA methylation of one of the alleles even though other mechanisms are possible [21]. POE could be missed in a standard GWAS of unrelated individuals, or the effect sizes could be underestimated where parental origin of alleles is not taken into account. Moreover, such POE could also provide insights into pleotropic relationships, which could only be seen when the parental origin of the alleles is considered. However, several studies have shown POE for complex traits, suggesting this to be a possible explanation for the sexual dimorphism and missing heritability of these traits [22,23,24,25,26,27]. An example of this is variants in KCNQ1, which showed an association to T2D in a large scale GWAS but also showed POE in families [28,29].

Given the previous observations of parent-of-origin effects on lipid traits, we selected 12 loci associated with lipid-related traits from genome-wide association studies (GWAS) published before 2012 to investigate if any of them could account for the parent of origin (POE) or sex-specific effects (i.e., sons and daughters) in families from the Botnia study. 

Dyslipidemia is strongly correlated with obesity. Obese individuals manifest elevated serum triglyceride, VLDL, apolipoprotein B, and non-HDL-C levels and are at an increased risk of developing cardiovascular disease [30]. Treatment for dyslipidemia is therefore often indicated for these patients. Given the strong relationship between lipid levels and obesity, we also investigated if the parental specific effects of the genetic variants were also observed on obesity measures in the families from two studies: the Botnia study and the HTB.

## 2. Materials and Methods

### 2.1. Study Cohorts

The Botnia Study was initiated in 1990. All patients with T2D visiting 5 health care centers in the Botnia region in western Finland and their families were invited to participate. Later on, these studies were extended to other parts of Finland and southern Sweden [17]. For this study, all individuals who were part of families where at least one parent and at least one child had participated in the study were selected, resulting in a total of 8066 individuals (offspring *n* = 3552) from 2257 families (Table 1).

The Hungarian Transdanubian Biobank (HTB) was initiated in 1992 at the Hungarian Heart Center in Balatonfüred. For this study, families with at least 1 parent and at least 1 offspring were selected, giving a total of 7884 individuals (offspring *n* = 3366) in 2264 families (Table 1).

We also tested for POE in unrelated individuals from the Diabetes Genetics Initiative (DGI) [31]; the Prevalence, Prediction, and Prevention of diabetes-Botnia study (PPP-Botnia) [32]; and Malmö Diet Cancer (MDC) [33] cohorts (for cohort characteristics, see Supplementary Table S1).

### 2.2. Phenotypes

Height and weight (in light, indoor clothing) were measured to the nearest cm and BMI calculated as (weight in kg)/(height in m^2^). Waist and hip circumference were measured with a non-elastic soft tape to the nearest cm, the waist halfway between the lowest rib and the iliac crest and hip as the widest part of the gluteal region. Blood samples were drawn after overnight fast for measurements of TC, HDL-C, triglycerides (TG) (using a Cobas Mira analyser (Hoffman–LaRoche, Basel, Switzerland)), and ApoA1, ApoA2 and ApoB (immunochemical assays (Orion Diagnostica, Espoo, Finland)), as previously described [17]. LDL-C was calculated using the Friedewald formula ((LDL-C = TC − HDL-C − TG/5) if TG < 400 mg/dL) [34]. The ratio between ApoB and ApoA1 was calculated as ApoB/ApoA1. For the HTB cohort, no lipid data were available.

Blom’s rank-based inverse normal transformation was applied to TG, APOB, and LDL values and all obesity measures. All the other plasma lipid level measurements were natural log transformed to achieve normal distribution. To remove outliers, values outside of 5 SD were removed; however, remaining data were all within 3 SD.

### 2.3. Genotyping

The study participants were genotyped using the Sequenom MassARRAY iPLEX Platform (Sequenom, San Diego, CA, USA) [35] for 172 SNPs already shown to be associated with diabetes and related traits. From this, 11 SNPs associated with lipids and fatty liver disease were selected (Supplementary Table S2). The SNPs were selected based on either showing additional association to other cardiometabolic traits and/or based on biological plausibility and interest. Additionally, the SNP rs4731702 in KLF14 were genotyped as a putative positive control using Taqman (Applied Biosystems, Waltham, MA, USA); due to failed genotyping in HTB, this SNP was only analysed for the Botnia cohort. 

The genotyping success rate was >90% for all the SNPs genotyped. A total of 4.8% of our samples were replicated and showed concordance with previous genotyping results, and the known genotyping controls showed concordance with expected results. All SNPs were in Hardy–Weinberg Equilibrium as tested in the individuals from the families.

### 2.4. Statistical Analyses

In the first model, Spearman rank correlation and partial correlation between father–offspring (son/daughter), mother–offspring (son/daughter), and father–mother was calculated including only the oldest child from each family. In the second model, all offspring from the families were included in a linear mixed model where family ID was used as random effects. A third model was computed similar to the second but with the addition of sex (when applicable), age, age^2^, and BMI of the offspring as covariates. 

To assess whether the difference between the paternal and maternal correlation coefficients were significant, Fisher r-to-z transformation was implemented. To assess the difference between the slopes from the linear mixed models, Wald’s test was applied.

Parental origin of alleles was inferred for all offspring. For heterozygous genotypes, parental origin of the allele was inferred by comparing with parental genotypes, i.e., ApBm or AmBp, where m was maternal and p was paternal. For the genetic analysis concerning lipid-related traits, families from the Botnia study were split into 2 random subsets, for discovery and replication, respectively (Supplementary Table S3). For a small subset of HTB offspring, we also had triglyceride levels, which were used as a second replication. 

To test for POE of genetic variants on the traits in the family-based cohorts, we performed a generalized estimating equation analysis using IBM SPSS Statistics 22 (IBM, Armonk, NY, USA), grouping by nuclear family ID. To determine if the allele manifested a paternal or maternal effect, we compared the effect of the minor allele inherited from the paternal side and from the maternal side independently, respectively. For the parent-of-origin effect analysis, we compared the effects of the paternal against the maternal minor allele in offspring carrying the heterozygous genotype. BMI, sex, age, age^2^, and diabetes affection status were included as covariates for all analyses. For replication, *p*-values of <0.05 with the same direction of effect were considered significant. Bonferroni corrected *p*-Values were indicated in the result tables.

For POE analyses in unrelated individuals, POE was implemented in QUICKTEST v0.98 (University of Lausanne, Lausanne, Switzerland) software onto mean genotypes, as described by Hoggart et al. [36]. The meta-analysis were performed using Metal v. 2011-03-25 (University of Michigan, Ann Arbor, MI, USA) [37].

## 3. Results

### 3.1. Parent-of-Origin Effects of Lipid Traits

Comparisons between generations in the Botnia study showed a statistically significant correlation between the father–offspring and mother–offspring lipid trait values (Supplementary Table S4). For all the traits, the correlation coefficient was larger between the mother–offspring pairs than the father–offspring pairs. The difference between maternal and paternal associations was significant for daughters but not for sons. The parent-of-origin effects were not significant when adjusted for sex and BMI.

To determine the genetic basis of these parental effects, locus-by-locus paternal, maternal, and parent-of-origin effects on plasma lipid levels were assessed for the selected loci in the discovery and replication sub-cohorts of the Botnia study. Seven SNPs showed nominal POE in the discovery sub-cohort (Table 2). Out of these SNP/Trait combinations, the parent-of-origin effect of the rs2131925 SNP at the *DOCK7/ANGPLT3* locus on ApoA2 levels was replicated in the replication subset.

In the combined analyses, a total of four SNPs showed a POE on at least one trait (Table 2). These included the rs2131925 SNP at the *DOCK7/ANGPLT3* locus, wherein the G allele was associated with decreased ApoA1 and ApoA2 when paternally inherited. However, the maternally inherited G allele was significantly associated with lower ApoB/ApoA1 ratio: the differences in the effect of the paternal and maternal G on ApoA1 and APoB/ApoA1 ratio were nominally significant, whereas the POE on ApoA2 was significant after correction for multiple testing. In addition to the rs2131925 SNP, the results included variants at *TMEM57* (rs12027135) wherein the A allele were associated with decreased ApoA2 levels when paternally inherited but increased ApoA2 levels when maternally inherited. The A allele of rs10503669 at the *LPL* locus was associated with decreased ApoA2 levels when maternally inherited. The rs12272004 SNP near the *APOA* gene also showed POE, with the A allele associated with decreasing ApoB and ApoB/ApoA1 ratio when paternally inherited but with increasing trait values when maternally inherited. The maternal A allele near the *APOA* gene was also associated with increased and the paternal allele with decreased TG levels; while the latter effect was not statistically significant, the POE was nominally significant. This POE was robustly replicated with the same direction of effect for the maternal and paternal alleles in the HTB families.

### 3.2. Sex-Specific POE

Levels of total cholesterol, HDL-C, TG, ApoA1, ApoB, and ApoB/Apo1 ratio showed stronger correlations between mothers and daughters compared to fathers and daughters, but no significant differences were seen between father–son and mother–son correlations (Supplementary Table S4). Therefore, the genetic basis of these POE was analysed in sons and daughters separately on lipids (Table 3). The ApoA1-lowering association of the paternal G allele and the POE of the rs2131925 SNP at the *DOCK7/ANGPTL3* locus was seen only in daughters but not in sons. The same paternal G allele was associated with decreased ApoA2 levels in sons as well as daughters; however, the POE was significant only in daughters. The paternal G allele also associated with ApoB/ApoA1 ratio in daughters in a POE manner; however, this association was not observed in sons. The POE of the variants at the *TMEM57* and *LPL* loci on ApoA2 levels, *APOB* and *KLF14* on ApoB/ApoA1 ratio, and *APOA* variant on TG levels were only seen in sons.

### 3.3. POE on Obesity Traits

Given the strong relationship between obesity and lipid levels, the selected SNPs were assessed for their POE on obesity traits, including body mass index (BMI), waist–hip ratio (WHR), waist–hip ratio adjusted for BMI (WHRadjBMI), and waist–height ratio (WHtR) (Table 4). The rs2131925 SNP at the *DOCK7/ANGPTL3* locus showed POE on WHR as well as WHRadjBMI in the families from the Botnia study, with the paternally inherited G allele associated with increased whereas maternally inherited G with decreased WHR and WHRadjBMI. The POE was also replicated in a second cohort of families from the HTB with directional consistency for the maternal and paternal allelic effects.

The A allele of the rs10503669 SNP at the *LPL* locus was associated with decreased WHR, WHRadjBMI, and WHtR when maternally inherited and showed the opposite effect for the paternal allele. Significant POE was seen for this variant for all the aforementioned obesity traits in the Botnia families. The meta-analysis POE *p*-values were significant for all the traits with directional consistency except for rs10503669 for WH when adjusting for BMI (Table 4).

This SNP also showed increased variance in obesity related traits in heterozygous individuals from three cohorts of unrelated individuals (DGI, PPP-Botnia, and MDC), further supporting our findings (Supplementary Table S5a,b).

### 3.4. Sex-Specific POE Effects on Obesity Traits

Given the sex-specific parental effects of lipid levels, the genetic sex-specific parental effects on obesity were next assessed. After meta-analysis of POE on obesity in daughters/sons from both studies, SNPs at three loci showed significant POE on obesity (Table 5). The *PABPC4* variant rs4660293 showed POE on WHR and WHRadjBMI only in the daughters, with the paternal allele associated with lower obesity measures. The LPL variant rs10503669 showed POE on BMI and WHR and WHtR in daughters, with the maternal A allele associated with lower BMI, WHR, and WHtR than its paternal counterpart in the Botnia and HTB families. A similar effect was also observed in sons in the Botnia study but not in HTB. The rs2131925 SNP at the *DOCK7/ANGPTL3* locus also showed POE on WHR and WHRadjBMI in daughters with the paternal G allele associated with increasing WHR and WHRadjBMI to a higher degree than the maternal G in both family cohorts. A similar trend was also seen in the Botnia sons before adjusting for BMI but not in HTB.

## 4. Discussion

Sex-specific parental effects of plasma lipid levels have been reported in multiple family studies, with the lipid levels showing a certain degree of heritability in the families. However, very few genetic variants have been shown to associate with lipid traits in a POE manner thus far. In the present study, we investigated the genetic basis of sex-specific parental effects of plasma lipid levels by examining if common variants associated with lipid levels from previous GWAS studies also associate with lipid traits in a POE-specific manner. Given the strong relationship between lipid traits and obesity, we assessed if these same variants showed POE on obesity.

We discovered genetic parental-specific associations for seven SNPs to at least one trait, including sex-specific POE. Our key findings were the robust POE of variants at the *LPL* and *DOCK7/ANGPTL3* loci on obesity measures in both family cohorts, and the same variants also showed POE on lipid traits in the Botnia family study. The variant at the LPL loci also showed potential POE in unrelated individuals.

In previous GWAS, the minor allele G of the rs2131925 SNP (ANGPTL3/DOCK7) associated with a decrease in TC, TG, and LDL-C levels [5,38], which would be beneficial in terms of cardiovascular health. Our study found POE on ApoA1, ApoA2, and ApoB/ApoA1 ratio, where the G allele, when inherited paternally, was associated with lower ApoA1 and ApoA2 but when maternally inherited, was associated with lower ApoB/ApoA1 ratio. When looking at obesity related traits, the G allele showed a trend to lower waist-hip ratio with and without adjusting for BMI when inherited maternally and increased ratio when inherited paternally in both Botnia and HTB. For all these traits, the direction of effect was such as would be beneficial for cardio-vascular health when the allele was maternally inherited. 

The SNP rs10503669 (*LPL*) showed a POE on ApoA2, with the maternally inherited A allele associated with decreased ApoA2. The A allele of this SNP has previously shown association to increased HDL and decreased TG and TC in previous GWAS. This SNP also showed POE for obesity traits, with the maternally inherited A allele associated with low-er WH, WHadjBMI, and WHtR in Botnia, while the paternally inherited A allele was associated to increased BMI and WHtR. The POE tests in HTB families did not reach significance, but the direction of effect showed the same trend, and the meta-analysis was significant for POE for BMI, WH, and WHtR. This was seen for both Botnia and HTB daughters (meta-analysis significant for BMI and WHtR) while only in Botnia sons and not for HTB sons. The maternal effect seen on ApoA2 would be expected to be negatively associated with cardiovascular, while the effect on obesity would be expected to be beneficial.

The *ANGPTL3* and *LPL* variants were also eQTLs for their respective genes (GTEx data [39]) and associated with ApoA2 levels. This is not surprising given that ApoA2 is the second largest component of HDL, and ANGPTL3 regulates plasma HDL through endothelial lipase (EL) [40], whereas LPL also contributes to HDL metabolism by facilitating FFA availability [30]. *ANGPTL3* is almost exclusively expressed in the liver and released into circulation, where it undergoes cleavage by hepatic proprotein convertases and is thus activated [41,42,43]. *ANGPTL3* is an important regulator of the lipoprotein lipase enzyme coding *LPL*, which is a key enzyme in the lipolysis of triglycerides, VLDLs, and chylomicrons [44,45,46]. Given the regulatory effects of *ANGPTL3* on *LPL*, the physiological consequences of the paternal and maternal effects on the lipid levels are then directionally consistent. 

The association to higher obesity measures of the paternal G allele of rs2131925 and the paternal A allele of rs10503669 was consistent in both the Botnia and the Hungarian cohorts. It is possible that this could be a representation of the kinship theory [47], with the paternal and maternal programming in direct oppositions and in a state of “tug-of-war,” manifested as maternal programming reducing obesity but paternal programming increasing obesity, further facilitated by the catch-up effect [48].

Previous studies have demonstrated the sexual dimorphism in lipid traits with some evidence for a genetic basis [5,49]. Consistent with previous studies, the present study showed a significant difference in correlation between father–offspring and mother–offspring for most of the traits tested. Genetic parent-of-origin effects of common variants could explain some of the parental discordance of phenotype correlations as well as sex-specific parental effects in lipid levels in offspring. Some recent studies have shown genetic differences between the genders, with SNPs either showing an association to a trait only in one gender or different effect sizes for men and women [5,50].

Analyses of gender-specific effect of genetic variants are possible to perform in unrelated groups. However, we also show that the picture is even more complex, with variants showing not only different effects in males vs. females but also that the parent-of-origin effect may differ between sons and daughters. This is something that would not be seen in unrelated individuals and may provide valuable clues to both the missing heritability of lipid traits as well as an explanation of some of the differences seen in lipid levels among the sexes.

Studies on POE are limited since family-based studies and especially investigation of POE require substantial study power. To address this, we selected a limited number of candidate genes to assess if they could explain some of the parental effects. Given the lack of replication for the lipid measurements in family data, we further subdivided the Botnia study into two random, family-based cohorts. Another limitation of our study regarding the variants showing sex-specific POE, with SNPs showing POE in daughters but not in sons, could be attributed to low study power or population specific effects. Another limitation of our study is that, due to low study power, very few SNPs showed genome-wide significant effects; however, the replication in another large family-based cohort (HTB) provides a robust validation of the findings. 

It is worth pointing out that our study does not investigate whether any of these SNPs is the functional variant driving these results, and we cannot prove a causal effect. Given the strong correlation between lipid levels and obesity, it could very well be that POE on obesity could be mediated by the POE on lipids or vice versa. However, large-scale studies are required to explore the causality through approaches like Mendelian randomization. 

Given the results, it is expected there could be a large number of genetic loci showing POE on lipid traits and obesity, and further studies in larger family cohorts will be necessary to determine the genetic basis of the sex-specific parental effects. Our results highlight the need to consider the possibility of POE in analyses of lipid traits and obesity in order to gain a correct understanding of their effects. A deeper knowledge of the genetic basis of dyslipidemia could help us understand the genes and pathways involved and lead to better ways of prevention, prediction, and treatment of CVD.

## Figures and Tables

**Table 1:** Cohort characteristics for the Botnia and HTB cohorts.

	**Botnia**											
	**All**		**Parents**		**Offspring**		**Parents/** **Offspring**	**Sons**		**Daughters**		**Sons/Daughters**
	**N**	**Mean ± SD**	**N**	**Mean ± SD**	**N**	**Mean ± SD**	***p***	**N**	**Mean ± SD**	**N**	**Mean ± SD**	***p***
N	8066		4514		3552			1782		1770		
N (male/female)	4039/4027		2257/2257		1782/1770							
Age (years)	5548	50.7 ± 16.6	2244	59.93 ± 12.52	3304	44.43 ± 16.09	<0.0001	1666	45.06 ± 16.47	1638	43.79 ± 15.68	0.02
BMI (kg/m^2^)	5546	26.69 ± 4.61	2244	27.2 ± 4.46	3302	26.34 ± 4.68	<0.0001	1666	26.54 ± 4.08	1636	26.13 ± 5.22	0.01
Waist/Hip Ratio	5332	0.90 ± 0.1	2135	0.91 ± 0.09	3197	0.89 ± 0.1	<0.0001	1615	0.94 ± 0.07	1582	0.84 ± 0.084	<0.0001
Waist/Height Ratio	5335	0.54 ± 0.08	2136	0.55 ± 0.07	3199	0.53 ± 0.078	<0.0001	1616	0.53 ± 0.07	1583	0.52 ± 0.08	0.0002
TC (mmol/L)	5295	5.44 ± 1.11	2119	5.7 ± 1.10	3176	5.27 ± 1.08	<0.0001	1607	5.30 ± 1.06	1569	5.25 ± 1.11	0.19
TG (mmol/L)	5292	1.47 ± 0.94	2119	1.57 ± 1.01	3173	1.41 ± 0.9	<0.0001	1604	1.52 ± 0.98	1569	1.29 ± 0.79	<0.0001
LDL-C (mmol/L)	5145	3.47 ± 0.99	2060	3.7 ± 0.98	3085	3.32 ± 0.96	<0.0001	1551	3.42 ± 0.95	1534	3.23 ± 0.97	<0.0001
HDL-C (mmol/L)	5172	1.31 ± 0.35	2072	1.30 ± 0.36	3100	1.32 ± 0.34	0.045	1566	1.20 ± 0.3	1534	1.43 ± 0.34	<0.0001
APOA1 (mg/L)	4636	135.88 ± 22.98	1866	136.57 ± 23.57	2770	135.42 ± 22.57	0.1	1404	128.40 ± 19.65	1366	142.63 ± 23.11	<0.0001
APOA2 (mg/L)	4372	36.06 ± 12.63	1793	35.75 ± 14.39	2579	36.27 ± 11.24	0.2	1316	36.73 ± 14.34	1263	35.80 ± 6.6	0.03
APOB (mg/L)	4623	93.39 ± 23.84	1862	97.86 ± 23.24	2761	90.37 ± 23.78	<0.0001	1401	93.79 ± 23.49	1360	86.84 ± 23.57	<0.0001
ApoB/ApoA1	4628	0.71 ± 0.22	1862	0.74 ± 0.22	2766	0.69 ± 0.22	<0.0001	1402	0.75 ± 0.22	1364	0.62 ± 0.20	<0.0001
**Affection Status**	**N**	**Valid Percent**	**N**	**Valid Percent**	**N**	**Valid Percent**		**N**	**Valid Percent**	**N**	**Valid Percent**	
Normal Glucose Tolerance	2608	47.01	873	38.90	1735	52.51		818	49.1	917	55.98	
Impaired Fasting Glucose	393	7.08	168	7.49	225	6.81		143	8.58	82	5.01	
Impaired Glucose Tolerance	514	9.26	215	9.58	299	9.05		121	7.26	178	10.87	
Type 2 Diabetes	1814	32.7	925	41.22	889	26.91		506	30.37	383	23.38	
Other, mostly T1D	196	3.53	55	2.27	149	4.39		75	4.32	74	4.46	
Missing	2533		2278		255			119		136		
	**HTB**											
	**All**		**Parents**		**Offspring**		**Parents/Offspring**	**Sons**		**Daughters**		**Sons/Daughters**
	**N**	**Mean ± SD**	**N**	**Mean ± SD**	**N**	**Mean ± SD**	***p***	**N**	**Mean ± SD**	**N**	**Mean ± SD**	***p***
N	7884		4518		3366			1548		1818		
N (Male/Female)	3807/4077				1548/1818							
Age (years)	6918	48.17 ± 16.82	3552	60.08 ± 11.79	3366	35.61 ± 11.29	<0.0001	1548	35.17 ± 11.25	1818	35.99 ± 11.31	0.04
BMI (kg/m^2^)	6918	27.89 ± 5.45	3552	29.23 ± 5.12	3366	26.48 ± 5.45	<0.0001	1548	27.39 ± 5.06	1818	25.70 ± 5.64	<0.0001
Waist/Hip Ratio	6852	0.86 ± 0.13	3487	0.87 ± 0.12	3365	0.85 ± 0.13	<0.0001	1548	0.95 ± 0.09	1817	0.76 ± 0.09	<0.0001
Waist/Height Ratio	6854	0.52 ± 0.09	3488	0.54 ± 0.088	3366	0.49 ± 0.08	<0.0001	1548	0.51 ± 0.07	1818	0.47 ± 0.09	<0.0001
**Affection Status**	**N**	**Valid Percent**	**N**	**Valid Percent**	**N**	**Valid Percent**		**N**	**Valid Percent**	**N**	**Valid Percent**	
Normal Glucose Tolerance	5119	74.0	2057	57.91	3062	90.97		1398	90.31	1664	91.53	
Impaired Glucose Tolerance	439	6.35	366	10.30	73	2.17		34	2.2	39	2.15	
Type 2 Diabetes	1345	19.44	1125	31.67	220	6.54		109	7.04	111	6.11	
Type 1 Diabetes	15	0.22	4	0.11	11	0.33		7	0.45	4	0.22	
Missing	966		966					0		0		

**Table 2:** Results of POE on lipid related traits.

Trait/SNP	Cohort	CHR	GENE	Location	E/O	N	B_MAT	P_MAT	B_PAT	P_PAT	P_POE
ApoA1 ^ln^											
rs2131925	discovery	1	*ANGTPL3/DOCK7*	intron	G/T	266	−0.002	0.89	−0.028	0.05	0.25
rs2131925	replication	1	*ANGTPL3/DOCK7*	intron	G/T	238	0.017	0.34	−0.027	0.046 *	0.03 *
rs2131925	combined	1	*ANGTPL3/DOCK7*	intron	G/T	504	0.006	0.6	−0.030	0.01 *	0.03 *
ApoA2 ^ln^											
rs12027135	discovery	1	*TMEM57*	intron	A/T	336	0.036	0.04 *	−0.041	0.02 *	0.001 *
rs12027135	replication	1	*TMEM57*	intron	A/T	278	0.028	0.12	0.016	0.37	0.64
rs12027135	combined	1	*TMEM57*	intron	A/T	614	0.035	0.01 *	−0.013	0.33	0.01 *
rs2131925	discovery	1	*ANGTPL3/DOCK7*	intron	G/T	252	−0.008	0.65	−0.064	0.0002 ^#^	0.02 *
rs2131925	replication	1	*ANGTPL3/DOCK7*	intron	G/T	222	0.02	0.31	−0.039	0.06	0.02 *
rs2131925	combined	1	*ANGTPL3/DOCK7*	intron	G/T	474	0.005	0.69	−0.053	0.0002 ^#^	0.001 *
rs10503669	discovery	8	*LPL*	intergenic	A/C	147	−0.085	0.0002 ^#^	−0.026	0.29	0.14
rs10503669	replication	8	*LPL*	intergenic	A/C	128	−0.041	0.09	0.037	0.11	0.11
rs10503669	combined	8	*LPL*	intergenic	A/C	275	−0.066	0.0002 ^#^	0.006	0.73	0.03 *
ApoB ^n^											
rs673548	discovery	2	*APOB*	intron	A/G	240	−0.162	0.10	0.117	0.17	0.02 *
rs673548	replication	2	*APOB*	intron	A/G	224	0.21	0.05	0.186	0.07	0.86
rs673548	combined	2	*APOB*	intron	A/G	464	0.022	0.76	0.152	0.02 *	0.15
rs12272004	discovery	11	*APOA*	intergenic	A/C	102	0.127	0.29	−0.444	0.004 *	0.002 *
rs12272004	replication	11	*APOA*	intergenic	A/C	102	0.045	0.75	0.017	0.92	0.89
rs12272004	combined	11	*APOA*	intergenic	A/C	204	0.077	0.42	−0.223	0.04 *	0.03 *
LDL-Cholesterol ^n^											
rs2479409	discovery	1	*PCSK9*	nearGene-5	G/A	327	0.020	0.83	−0.223	0.02 *	0.04 *
rs2479409	replication	1	*PCSK9*	nearGene-5	G/A	325	−0.084	0.36	−0.14	0.11	0.62
rs2479409	combined	1	*PCSK9*	nearGene-5	G/A	652	−0.03	0.64	−0.178	0.01 *	0.07
Ratio ApoB/ApoA1 ^ln^											
rs10503669	discovery	8	*LPL*	intergenic	A/C	153	−0.096	0.01 *	0.006	0.86	0.04 *
rs10503669	replication	8	*LPL*	intergenic	A/C	132	0.004	0.91	0.004	0.91	0.74
rs10503669	combined	8	*LPL*	intergenic	A/C	285	−0.053	0.06	0.004	0.88	0.24
rs12272004	discovery	11	*APOA*	intergenic	A/C	102	0.037	0.34	−0.112	0.01 *	0.01 *
rs12272004	replication	11	*APOA*	intergenic	A/C	102	0.034	0.41	−0.03	0.52	0.41
rs12272004	combined	11	*APOA*	intergenic	A/C	204	0.033	0.26	−0.074	0.01 *	0.01 *
rs2131925	discovery	1	*ANGTPL3/DOCK7*	intron	G/T	266	−0.023	0.4	0.017	0.49	0.33
rs2131925	replication	1	*ANGTPL3/DOCK7*	intron	G/T	238	−0.074	0.02 *	0.036	0.22	0.01 *
rs2131925	combined	1	*ANGTPL3/DOCK7*	intron	G/T	504	−0.046	0.03 *	0.029	0.14	0.01 *
Triglycerides ^n^											
rs12272004	discovery	11	*APOA*	intergenic	A/C	109	0.188	0.12	−0.203	0.24	0.06
rs12272004	replication	11	*APOA*	intergenic	A/C	110	0.228	0.06	−0.023	0.89	0.2
rs12272004	combined	11	*APOA*	intergenic	A/C	219	0.223	0.01 *	−0.092	0.43	0.03 *
rs12272004	*HTB*	11	*APOA*	intergenic	A/C	13	0.727	0.0001 ^#^	−0.313	0.26	0.0001 ^#^

CHR, chromosome; GENE, nearest gene; E/O, effect allele/other allele; N, number of heterozygous offspring tested; B_MAT, B_PAT, effect of maternally/paternally inherited minor allele compared to major homozygous allele carriers; P_MAT, *p*-Value for maternal effect; P_PAT, *p*-Value for paternal effect; P_POE, *p*-Value for parent-of-origin effect; ^ln^, trait was naturally log transformed; ^n^, trait was normalized using Blom’s rank-based inverse normal transformation. All analyses were adjusted for sex, BMI, age, age^2^, and diabetes affection status. * *p* < 0.05, ^#^ *p* < 0.0005 (Bonferroni corrected *p*-value).

**Table 3:** POE on lipid levels in sons and daughters separately in Botnia.

TRAIT/SNP	CHR	GENE	Location	E/O	B_MAT	P_MAT	B_PAT	P_PAT	P_POE		B_MAT	P_MAT	B_PAT	P_PAT	P_POE
					Sons						Daughters				
ApoA1 ^ln^															
rs2131925	1	*ANGTPL3/DOCK7*	intron	G/T	−0.006	0.74	−0.028	0.05	0.21		0.018	0.28	−0.028	0.05 *	0.03 *
ApoA2 ^ln^															
rs12027135	1	*TMEM57*	intron	A/T	0.03	0.07	−0.02	0.25	0.02 *		0.029	0.14	−0.017	0.35	0.07
rs2131925	1	*ANGTPL3/DOCK7*	intron	G/T	−0.005	0.81	−0.05	0.01 *	0.1		0.016	0.36	−0.043	0.01 *	0.01 *
rs10503669	8	*LPL*	intergenic	A/C	−0.056	0.01 *	0.025	0.25	0.04 *		−0.071	0.001 *	−0.017	0.49	0.35
ApoB ^n^															
rs12272004	11	*APOA*	intergenic	A/C	0.145	0.26	−0.193	0.25	0.1		0.061	0.63	−0.27	0.07	0.07
Ratio ApoB/ApoA1 ^ln^															
rs2131925	1	*ANGTPL3/DOCK7*	intron	G/T	−0.046	0.13	−0.012	0.65	0.33		−0.034	0.24	0.07	0.01 *	0.01 *
rs673548	2	*APOB*	intron	A/G	−0.001	0.98	0.072	0.002 *	0.03 *		0.023	0.37	−0.006	0.86	0.58
rs4731702	7	*KLF14*	intergenic	T/C	−0.059	0.02 *	0.008	0.79	0.03 *		0.013	0.65	0	0.99	0.88
rs12272004	11	*APOA*	intergenic	A/C	0.034	0.42	−0.057	0.21	0.17		0.028	0.47	−0.09	0.04 *	0.07
Triglycerides ^n^															
rs12272004	11	*APOA*	intergenic	A/C	0.344	0.01 *	−0.104	0.47	0.02 *		0.098	0.34	−0.069	0.71	0.43

CHR, chromosome; GENE, nearest gene; E/O, effect allele/other allele; B_MAT, B_PAT, effect of maternally/paternally inherited minor allele compared to major homozygous allele carriers; P_MAT, *p*-Value for maternal effect; P_PAT, *p*-Value for paternal effect; P_POE, *p*-Value for parent-of-origin effect; ^ln^, trait was naturally log transformed; ^n^, trait was normalized using Blom’s rank-based inverse normal transformation. All analyses were adjusted for BMI, age, age^2^, and diabetes affection status. * *p* < 0.05; Bonferroni corrected *p*-Value < 0.0005.

**Table 4:** POE on obesity related traits in Botnia and HTB.

TRAIT/SNP	CHR	GENE	LOCATION	E/O	N	B_MAT	P_MAT	B_PAT	P_PAT	P_POE		N	B_MAT	P_MAT	B_PAT	P_PAT	P_POE	Meta-Analysis *p*-Value
					**Botnia**							**HTB Families**						
BMI																		
rs4731702	7	*KLF14*	intergenic	T/C	643	−0.105	0.11	0.045	0.47	0.04 *								
rs10503669	8	*LPL*	intergenic	A/C	306	−0.098	0.22	0.257	0.001 *	0.001 *		266	0.053	0.61	0.155	0.07	0.44	0.004*
WHR																		
rs2131925	1	*ANGPTL3/DOCK7*	intron	G/T	576	−0.101	0.17	0.121	0.11	0.02 *		719	−0.075	0.22	0.097	0.12	0.03 *	0.006 *
rs10503669	8	*LPL*	intergenic	A/C	306	−0.337	0.001 *	0.09	0.26	0.001 *		266	0.071	0.55	0.139	0.07	0.63	0.005 *
WHRadjBMI																		
rs2131925	1	*ANGTPL3/DOCK7*	intron	G/T	576	−0.132	0.08	0.073	0.32	0.03 *		719	−0.026	0.68	0.132	0.02 *	0.04 *	0.009 *
rs10503669	8	*LPL*	intergenic	A/C	306	−0.316	0.001 *	−0.02	0.81	0.02 *		266	0.041	0.72	0.053	0.48	0.92	0.09
WHtR																		
rs10503669	8	*LPL*	intergenic	A/C	306	−0.235	0.01 *	0.317	0.00004 ^#^	0.000001 ^#^		266	−0.025	0.81	0.154	0.07	0.17	0.000003 ^#^

CHR, chromosome; GENE, nearest gene; E/O, effect allele/other allele; B_MAT, B_PAT, effect of maternally/paternally inherited minor allele compared to major homozygous allele carriers; N, number of heterozygous offspring tested; P_MAT, *p*-Value for maternal effect; P_PAT, *p*-Value for paternal effect; P_POE, *p*-Value for parent-of-origin effect. All traits was normalized using Blom’s rank-based inverse normal transformation, and adjusted for sex, age, age^2^, and diabetes affection status. * *p* < 0.05, ^#^ *p* < 0.001 (Bonferroni corrected *p*-Value).

**Table 5:** Sex-specific POE effects on obesity traits.

TRAIT/SNP	CHR	GENE	SNP location	E/O	B_MAT	P_MAT	B_PAT	P_PAT	P_POE		B_MAT	P_MAT	B_PAT	P_PAT	P_POE	Meta_P_POE
					**Botnia—Daughters**						**HTB—Daughters**					
BMI																
rs10503669	8	LPL	intergenic	A/C	−0.273	0.01 *	0.195	0.1	0.002 *		−0.038	0.84	0.367	0.001 *	0.05	0.001 *
WHR																
rs2131925	1	ANGTPL3/ DOCK7	intron	G/T	0.064	0.51	0.219	0.04 *	0.24		−0.152	0.06	0.07	0.41	0.04 *	0.05
rs4660293	1	PABPC4	intron	G/A	0.098	0.28	−0.073	0.4	0.15		0.179	0.03 *	−0.114	0.11	0.003 *	0.004 *
rs10503669	8	LPL	intergenic	A/C	−0.379	0.01 *	0.103	0.34	0.01 *		0.093	0.54	0.299	0.001 *	0.23	0.02 *
WHRadjBMI																
rs2131925	1	ANGTPL3/ DOCK7	intron	G/T	0.003	0.98	0.21	0.05 *	0.12		−0.095	0.24	0.128	0.1	0.03 *	0.02 *
rs4660293	1	PABPC4	intron	G/A	0.102	0.28	−0.131	0.15	0.05		0.094	0.26	−0.175	0.01 *	0.01 *	0.004 *
WHtR																
rs10503669	8	LPL	intergenic	A/C	−0.326	0.01 *	0.302	0.004 *	0.0001 ^#^		−0.022	0.88	0.347	0.001 *	0.03 *	0.00004 ^#^
					**Botnia—Sons**						**HTB—Sons**					
BMI																
rs10503669	8	LPL	intergenic	A/C	0.081	0.44	0.332	0.002 *	0.08		0.11	0.38	−0.151	0.25	0.14	0.06
WHR																
rs2131925	1	ANGTPL3/ DOCK7	intron	G/T	−0.253	0.01 *	−0.004	0.96	0.04 *		0.021	0.82	0.105	0.24	0.47	0.09
rs4660293	1	PABPC4	intron	G/A	0.016	0.86	0.048	0.57	0.78		0.007	0.94	0.023	0.77	0.88	0.94
rs10503669	8	LPL	intergenic	A/C	−0.318	0.02 *	0.077	0.51	0.02 *		0.042	0.79	−0.025	0.84	0.74	0.08
WHRadjBMI																
rs2131925	1	ANGTPL3/ DOCK7	intron	G/T	−0.232	0.02 *	−0.143	0.11	0.47		0.068	0.46	0.13	0.13	0.59	0.63
rs4660293	1	PABPC4	intron	G/A	0.049	0.58	0.144	0.07	0.4		−0.009	0.91	0.093	0.24	0.31	0.38
WHtR																
rs10503669	8	LPL	intergenic	A/C	−0.145	0.22	0.347	0.002 *	0.002 *		−0.057	0.68	−0.131	0.31	0.69	0.01 *

Parent-of-origin effects on obesity related traits in sons. CHR, chromosome; GENE, nearest gene; E/O, effect allele/other allele; B_MAT, B_PAT, effect of maternally/paternally inherited effect allele compared to major homozygous for other allele; P_MAT, *p*-Value for maternal effect; P_PAT, *p*-Value for paternal effect; P_POE, *p*-Value for parent-of-origin effect. All traits was normalized using Blom’s rank-based inverse normal transformation, and adjusted for age, age^2^, and diabetes affection status. * *p* < 0.05, ^#^ *p* < 0.001 (Bonferroni corrected *p*-Value).

## Data Availability

All data related to genotypes and phenotypes are deposited at the LUDC repository (www.ludc.lu.se/resources/repository, accessed on 15 September 2021) under the following accession numbers and are available upon request: LUDC2021.10.1 (Botnia study), LUDC2021.10.2 (Hungarian transdanubian biobank), LUDC2021.10.3 (PPP Botnia), LUDC2021.10.4 (DGI), LUDC2021.10.5 (MDC).

## Supplementary Materials

The following are available online at https://www.mdpi.com/article/10.3390/genes13010091/s1: Supplementary Table S1, Cohort characteristics for the Botnia cohort, split randomly into two sub-cohorts for discovery and replication; Supplementary Table S2, SNPs selected from the ANDIS panel that had previously shown association to lipid related traits; Supplementary Table S3, Cohort characteristics for PPP, DGI, and MDC cohorts; Supplementary Table S4, Correlations between parent and offspring lipid trait values; Supplementary Table S5, Replication PPP/DGI/MDC results.
