# Supplementary material for: Lipid-Associated Variants near ANGPTL3 and LPL Show Parent-of-Origin Specific Effects on Blood Lipid Levels and Obesity
Source: Genes (Basel). 2021 Dec 29;13(1):91. doi: 10.3390/genes13010091 (PMC8774740; doi:10.3390/genes13010091)
Supplement: Supplementary file 1 [file genes-13-00091-s001.zip › LipidManuscript_supplementary_Table S1.pdf]

Supplementary Table S1. Cohort characteristics for PPP-Botnia, DGI and MDC.

	PPP-Botnia		DGI		MDC	
	**N**	**Mean ± SD**	**N**	**Mean ± SD**	**N**	**Mean ± SD**
N	5208		3142		3311	
N Male/Female	2443/2765		1533/1609		1340/1971	
Age	5208	49.61 ± 15.69	3129	61.73 ± 10.67	3311	72.0 ± 6.0
BMI	5205	26.51± 4.45	3111	27.62 ± 4.21	3309	25.70 ± 2.21
WH	5188	0.88 ± 0.1	2346	0.92 ± 0.09	1743	0.86 ± 0.09
WhtR	5189	0.53 ± 0.07	2991	0.56± 0.07	1743	0.50 ± 0.06
**Affection status**	**N**	**Valid percent**	**N**	**Valid percent**	**N**	**Valid percent**
Control	4854	93.22	1525	48.54	2974	90.0
Case	324	6.22	1617	51.46	337	10.0
Missing	30	0.58				

BMI; Body Mass index, WH; waist/Hip ratio, WHtR; Waist/Height ratio, Control; Not diabetes mellitus, Case; Type 2 Diabetes.
