# Supplementary material for: Lipid-Associated Variants near ANGPTL3 and LPL Show Parent-of-Origin Specific Effects on Blood Lipid Levels and Obesity
Source: Genes (Basel). 2021 Dec 29;13(1):91. doi: 10.3390/genes13010091 (PMC8774740; doi:10.3390/genes13010091)
Supplement: Supplementary file 1 [file genes-13-00091-s001.zip › LipidManuscript_supplementary_Table S2.pdf]

Supplementary Table S2. SNPs selected from previous GWAS with significant association with lipid related traits.

SNP	CHR	Gene/ nearest Gene	GENE NAME	SNP location	E/O	Trait	Other associations
rs12027135	1	*TMEM57*	transmembrane protein 57	intron	A/T	TC (− , LDL (−)	Height
rs2131925	1	*DOCK7 / ANGTPL3*	dedicator of cytokinesis 7	intron	G/T	TG (−), TC (−), LDL (−)	
rs2479409	1	*PCSK9*	proprotein convertase subtilisin/kexin type 9	nearGene-5	G/A	LDL (+), TC (+)	
rs4660293	1	*PABPC4*	poly(A) binding protein, cytoplasmic 4	intron	G/A	HDL (−)	T2D, BMI, (nom: WHR, height)
rs629301	1	*CELSR2*	cadherin, EGF LAG seven-pass G-type receptor 2	coding – UTR3	A/C	LDL (+), TC (+), ApoB (+)	Height
rs7515577	1	*EVI5*	ecotropic viral integration site 5	intron	C/A	TC (−)	BMI
rs673548	2	*APOB*	apolipoprotein B	intron	G/A	TG (+), HDL (−), VLDL (+)	
rs6756629	2	*ABCG5*	ATP-binding cassette, sub-family G (WHITE), member 5	coding - missense	G/A	LDL (+), TC(+)	CAD
rs10503669	8	*LPL*	lipoprotein lipase	intergenic	A/C	HDL(+), TG (−), TC (−)	T2D, CAD, WHRadjBMI
rs12272004	11	*APOA*	lipoprotein, Lp(a)	intergenic	C/A	TG (−), LDL (−),TC (−)	CAD
rs738409	22	*PNPLA3*	patatin-like phospholipase domain containing 3 / adiponutrin	coding - missense	G/C	Increasing risk of fatty liver/Hepatic steatosis / Obesity/Liver enzyme levels	T2D
rs4731702	7	*KLF14*	Kruppel-Like Factor 14	intergenic	T/C	HDL (+), T2D (−)	T2D, BMI, BP, WHRadjBMI

Chr: Chromosome E/O: Effect allele, other allele. Trait: Traits for which the SNP has shown association, with direction of effect (+ or −) for each trait.
