# Supplementary material for: Lipid-Associated Variants near ANGPTL3 and LPL Show Parent-of-Origin Specific Effects on Blood Lipid Levels and Obesity
Source: Genes (Basel). 2021 Dec 29;13(1):91. doi: 10.3390/genes13010091 (PMC8774740; doi:10.3390/genes13010091)
Supplement: Supplementary file 1 [file genes-13-00091-s001.zip › LipidManuscript_supplementary_Table S3.pdf]

Supplementary Table S3. Cohort characteristics for the Botnia cohort, split randomly into two sub-cohorts for discovery & replication.

	Discovery sample		Replication sample	
	**N**	**Mean ± SD**	**N**	**Mean ± SD**
N	1822		1730	
N(male/female)	915/907		867/863	
Age (years)	1624	43.91 ± 15.75	1527	44.69 ± 16.27
BMI (kg/m^2)	1624	26.42 ± 4.74	1527	26.22 ± 4.65
Waist/Hip Ratio	1591	0.89 ± 0.09	1508	0.89 ± 0.1
Waist/Height Ratio	1593	0.53 ± 0.08	1508	0.52 ± 0.08
CHOL (mmol/L)	1622	5.28 ± 1.1	1524	5.27 ± 1.07
TRIGL (mmol/L)	1621	1.41 ± 0.86	1522	1.41 ± 0.92
LDL (mmol/L)	1590	3.33 ± 0.97	1493	3.32 ± 0.96
HDL (mmol/L)	1595	1.31 ± 0.33	1503	1.32 ± 0.35
APOA1 (mg/l)	1404	135.49 ± 22.93	1336	135.16 ± 22.14
APOA2 (mg/l)	1306	36.28 ± 6.73	1249	35.84 ± 6.73
APOB (mg/l)	1400	90.75 ± 24.40	1331	89.99 ± 23.17
ApoB/ApoA1	1402	0.69 ± 0.23	1334	0.69 ± 0.22
**Affection status**	**N**	**Valid Percent**	**N**	**Valid Percent**
Normal Glucose Tolerance	896	55.17	796	52.13
Impaired Fasting Glucose	103	6.34	120	7.86
Impaired Glucose Tolerance	147	9.05	147	9.63
Type 2 Diabetes	423	26.05	404	26.46
Other, mostly T1D	55	3.26	60	3.86
Missing	198		203	
