# Supplementary material for: Lipid-Associated Variants near ANGPTL3 and LPL Show Parent-of-Origin Specific Effects on Blood Lipid Levels and Obesity
Source: Genes (Basel). 2021 Dec 29;13(1):91. doi: 10.3390/genes13010091 (PMC8774740; doi:10.3390/genes13010091)
Supplement: Supplementary file 1 [file genes-13-00091-s001.zip › LipidManuscript_supplementary_Table S4a&b.pdf]

Supplementary Table S4a. Spearman correlation between the fathers-offspring and mothers-offspring lipid trait values to only oldest offspring, sons and daughters.

TRAIT	EST	SE	P	EST	SE	P	EST	SE	P	N	Z	P_DIFF
	Father-offspring			Mother-offspring			Mother-father					
Oldest offspring												
APOA1	0.150	0.037	0.0001	0.207	0.037	<0.0001	-0.002	0.036	0.950	619	1.025	0.153
APOA2	0.159	0.042	0.0002	0.249	0.041	<0.0001	-0.036	0.038	0.346	581	1.558	0.06
APOB	0.109	0.033	0.0012	0.155	0.035	<0.0001	-0.017	0.032	0.597	615	0.817	0.207
ApoB/ApoA1	0.101	0.032	0.0015	0.204	0.032	<0.0001	-0.011	0.030	0.713	614	1.813	0.035 #
CHOL	0.193	0.032	<0.0001	0.167	0.033	<0.0001	-0.058	0.031	0.062	732	-0.483	0.315
HDLCHOL	0.111	0.031	0.0004	0.204	0.031	<0.0001	0.016	0.028	0.581	713	1.794	0.036 #
TRIGL	0.054	0.032	0.0950	0.197	0.033	<0.0001	0.023	0.032	0.479	731	2.797	0.003 #
Oldest daughters												
APOA1	0.151	0.056	0.0072	0.231	0.054	<0.0001	0.023	0.052	0.656	324	1.057	0.145
APOA2	0.151	0.062	0.0163	0.218	0.059	0.0003	-0.070	0.057	0.215	304	0.814	0.208
APOB	0.094	0.048	0.0506	0.146	0.049	0.0033	0.024	0.046	0.598	323	0.666	0.253
ApoB/ApoA1	0.126	0.044	0.0048	0.190	0.045	<0.0001	0.033	0.041	0.420	323	0.841	0.2
CHOL	0.164	0.044	0.0002	0.124	0.048	0.0105	-0.038	0.045	0.396	386	-0.549	0.292
HDLCHOL	0.081	0.043	0.0575	0.233	0.044	<0.0001	0.010	0.044	0.825	378	2.125	0.017 #
TRIGL	0.097	0.041	0.0200	0.179	0.043	<0.0001	0.031	0.041	0.450	388	1.187	0.118
Oldest sons												
APOA1	0.154	0.049	0.0020	0.186	0.051	0.0003	-0.032	0.050	0.525	295	0.399	0.345
APOA2	0.176	0.055	0.0016	0.272	0.056	<0.0001	-0.013	0.050	0.802	277	1.152	0.125
APOB	0.118	0.045	0.0094	0.151	0.048	0.0019	-0.029	0.043	0.503	292	0.395	0.346
ApoB/ApoA1	0.068	0.045	0.1301	0.204	0.047	<0.0001	-0.045	0.045	0.313	291	1.618	0.053
CHOL	0.216	0.047	<0.0001	0.194	0.046	<0.0001	-0.066	0.043	0.126	346	-0.299	0.382
HDLCHOL	0.148	0.047	0.0017	0.180	0.045	0.0001	0.024	0.038	0.529	335	0.418	0.338
TRIGL	0.014	0.049	0.7777	0.213	0.051	<0.0001	0.025	0.049	0.609	343	2.671	0.004 #

EST; Estimates SE; standard error P; p-value P_DIFF; p-value for differences in correlation between mother-offspring and father-offspring. No covariates. # p <0.05

Supplementary Table S4b. Correlations between the father-offspring and mother-offspring lipid trait values to all offspring, sons and daughters using linear mixed models and different adjustments.

TRAIT	EST	SE	P	N	EST	SE	P	N	P_DIFF
	Father-offspring				Mother-offspring				
All Offspring									
ApoA1	0.195	0.031	<0.0001	1328	0.274	0.030	<0.0001	1330	0.06
ApoA2	0.207	0.031	<0.0001	1272	0.225	0.030	<0.0001	1275	0.67
ApoB	0.141	0.032	<0.0001	1321	0.226	0.033	<0.0001	1325	0.045 [#]
ApoB/ApoA1	0.183	0.032	<0.0001	1316	0.282	0.031	<0.0001	1324	0.02 [#]
Chol	0.193	0.029	<0.0001	1521	0.237	0.030	<0.0001	1524	0.3
HDL	0.204	0.030	<0.0001	1501	0.265	0.028	<0.0001	1499	0.12
TRIG	0.139	0.029	<0.0001	1522	0.236	0.030	<0.0001	1523	0.01 [#]
Daughters									
ApoA1	0.162	0.043	0.0002	660	0.254	0.039	<0.0001	663	0.11
ApoA2	0.203	0.044	<0.0001	630	0.207	0.042	<0.0001	632	0.94
ApoB	0.115	0.041	0.005	656	0.226	0.041	<0.0001	662	0.05
ApoB/ApoA1	0.185	0.041	<0.0001	655	0.288	0.039	<0.0001	663	0.05
Chol	0.165	0.036	<0.0001	760	0.243	0.038	<0.0001	768	0.06
HDL	0.131	0.037	0.0005	749	0.261	0.034	<0.0001	754	0.01 [#]
TRIG	0.114	0.037	0.002	760	0.206	0.035	<0.0001	767	0.07
Sons									
ApoA1	0.210	0.037	<0.0001	668	0.286	0.037	<0.0001	667	0.14
ApoA2	0.208	0.038	<0.0001	642	0.240	0.038	<0.0001	643	0.14
ApoB	0.164	0.042	0.0001	665	0.235	0.045	<0.0001	663	0.25
ApoB/ApoA1	0.182	0.041	<0.0001	661	0.276	0.041	<0.0001	661	0.1
Chol	0.236	0.041	<0.0001	761	0.256	0.041	<0.0001	756	0.73
HDL	0.236	0.037	<0.0001	752	0.258	0.035	<0.0001	745	0.65
TRIG	0.143	0.041	0.0006	762	0.262	0.044	<0.0001	756	0.048 [#]
All Offspring									
ApoA1*	0.188	0.030	<0.0001	1316	0.244	0.028	<0.0001	1319	0.18
ApoA2*	0.212	0.032	<0.0001	1261	0.230	0.031	<0.0001	1264	0.69
ApoB/ApoA1*	0.138	0.027	<0.0001	1304	0.195	0.026	<0.0001	1313	0.13
Chol*	0.208	0.025	<0.0001	1507	0.170	0.028	<0.0001	1511	0.32
HDLCHOL*	0.166	0.026	<0.0001	1487	0.222	0.024	<0.0001	1486	0.11
TRIG*	0.111	0.027	<0.0001	1508	0.164	0.027	<0.0001	1510	0.16
All Daughters									
ApoA1*	0.154	0.043	0.0004	657	0.236	0.039	<0.0001	660	0.16
ApoA2*	0.187	0.045	<0.0001	627	0.224	0.041	<0.0001	629	0.55
ApoB/ApoA1*	0.155	0.037	<0.0001	652	0.214	0.036	<0.0001	660	0.25
Chol*	0.183	0.033	<0.0001	755	0.198	0.037	<0.0001	763	0.76
HDLCHOL*	0.111	0.035	0.002	744	0.227	0.032	<0.0001	749	0.02 [#]
TRIG*	0.093	0.034	0.007	755	0.162	0.034	<0.0001	762	0.15

All sons									
ApoA1*	0.215	0.036	<0.0001	659	0.252	0.036	<0.0001	659	0.46
ApoA2*	0.218	0.039	<0.0001	634	0.234	0.039	<0.0001	635	0.78
ApoB/ApoA1*	0.119	0.034	0.0005	652	0.168	0.035	<0.0001	653	0.31
Chol*	0.223	0.034	<0.0001	752	0.167	0.036	<0.0001	748	0.26
HDLCHOL*	0.216	0.035	<0.0001	743	0.216	0.032	<0.0001	737	1.00
TRIG*	0.111	0.037	0.003	753	0.175	0.040	<0.0001	748	0.24

EST; Estimates SE; standard error P; p-value P_DIFF; p-value for differences in correlation between mother-offspring and father-offspring. * Adjusted for age. sex and BMI. # p <0.05
