# Supplementary material for: Lipid-Associated Variants near ANGPTL3 and LPL Show Parent-of-Origin Specific Effects on Blood Lipid Levels and Obesity
Source: Genes (Basel). 2021 Dec 29;13(1):91. doi: 10.3390/genes13010091 (PMC8774740; doi:10.3390/genes13010091)
Supplement: Supplementary file 1 [file genes-13-00091-s001.zip › LipidManuscript_supplementary_Table S5a&b.pdf]

Supplementary Table S5a. Parent-of-origin test in unrelated individuals from DGI, MDC and PPP-Botnia.

TRAIT	SNP	A	B	meanAA	meanAB	meanBB	rSqHat	varAA	varAB	varBB	beta	se	p-value
DGI													
BMI	rs10503669	A	C	32.8	488.2	2440.0	0.9770	0.871	1.032	0.995	0.013	0.030	0.331
WH	rs10503669	A	C	23.6	344.6	1835.8	0.9750	0.999	0.960	1.008	-0.033	0.034	0.838
WHtR	rs10503669	A	C	31.7	465.6	2358.7	0.9772	0.779	1.025	0.998	0.002	0.032	0.479
MDC													
BMI	exm-rs10503669	A	C	63.0	1322.3	6924.7	0.9998	1.243	1.107	0.978	0.056	0.019	0.002 #
WH	exm-rs10503669	A	C	63.0	1323.3	6935.7	0.9998	0.967	1.056	0.990	0.034	0.016	0.015 #
WHtR	exm-rs10503669	A	C	63.0	1323.3	6935.7	0.9998	1.048	1.073	0.986	0.048	0.018	0.004 #
PPP-Botnia													
BMI	exm-rs10503669	A	C	33.0	749.2	3604.8	0.9998	1.442	0.966	1.003	0.000	0.024	0.506
WH	exm-rs10503669	A	C	33.0	744.2	3592.8	0.9998	1.018	0.963	1.006	-0.010	0.023	0.673
WHtR	exm-rs10503669	A	C	33.0	754.2	3593.8	0.9998	1.395	0.947	1.0068	-0.0157	0.024	0.740
DGI													
BMI ^	rs10503669	A	C	32.8	488.2	2440.0	0.9770	0.890	1.028	0.996	0.012	0.030	0.349
WHR ^*	rs10503669	A	C	23.6	345.6	1840.8	0.9751	0.911	1.008	0.998	0.019	0.035	0.292
WHtR ^*	rs10503669	A	C	31.7	465.6	2357.7	0.9772	0.814	1.049	0.993	0.008	0.031	0.397
MDC													
BMI ^	exm-rs10503669	A	C	63.0	1322.3	6924.7	0.9998	1.274	1.102	0.978	0.052	0.020	0.004 #
WH ^*	exm-rs10503669	A	C	63.0	1323.3	6935.7	0.9998	1.095	1.031	0.993	0.010	0.019	0.307
WHtR ^*	exm-rs10503669	A	C	63.0	1323.3	6935.7	0.9998	1.135	1.057	0.988	0.037	0.020	0.031 #
PPP-Botnia													
BMI ^	exm-rs10503669	A	C	33.0	749.2	3604.8	0.9998	1.469	0.972	1.002	0.000	0.024	0.492
WH ^*	exm-rs10503669	A	C	33.0	745.2	3592.8	0.9998	1.228	0.980	1.000	-0.013	0.024	0.707
WHtR ^*	exm-rs10503669	A	C	33.0	745.2	3593.8	0.9998	1.395	0.947	1.007	-0.016	0.024	0.742

POE was implemented in QUICKTEST onto mean genotypes. Adjusted for age and diabetes affection status. ^ also adjusted for sex. *also adjusted for BMI # p <0.05

Supplementary Table S5b. Meta-analysis of parent-of-origin test in DGI, MDC and PPP-Botnia

TRAIT	SNP	Allele1	Allele2	Freq1	FreqSE	MinFreq	MaxFreq	Weight	Zscore	Direction	P-value
BMI [¤]	rs10503669	A	C	0.09	0.003	0.0871	0.0935	15658.01	3.023	+ + +	0.0025 [#]
WH*	rs10503669	A	C	0.089	0.0025	0.0871	0.0928	14903	2.012	+ + −	0.04425 [#]
WHtR*	rs10503669	A	C	0.0897	0.0028	0.0871	0.0928	15549	2.292	+ + −	0.02191 [#]
BMI	rs10503669	A	C	0.09	0.003	0.0871	0.0935	15658.01	2.323	+ + −	0.0202 [#]
WH ^	rs10503*669	A	C	0.089	0.0025	0.0871	0.0927	14896	1.502	− + −	0.1331
WHtR ^	rs10503669	A	C	0.0897	0.0028	0.0871	0.0928	15550	2.221	+ + −	0.02635 [#]

All analyses adjusted for age and diabetes affection status ¤ also adjusted for sex *also adjusted for sex and bmi. ^ Also adjusted for bmi. [#] p <0.05
